# Development and validation of machine learning-based models for predicting healthcare-associated bacterial/fungal infections among COVID-19 inpatients: a retrospective cohort study

**DOI:** 10.1186/s13756-024-01392-7

**Published:** 2024-04-14

**Authors:** Min Wang, Wenjuan Li, Hui Wang, Peixin Song

**Affiliations:** 1 Department of Infection Management, Nanjing Drum Tower Hospital, Affiliated Hospital of Medical School,Nanjing University, 321 Zhongshan Road, Nanjing, Jiangsu Province 210009 China; 2 Department of Medical Big Data, Nanjing Drum Tower Hospital, Affiliated Hospital of Medical School, Nanjing University, 321 Zhongshan Road, Nanjing, Jiangsu Province 210009 China

**Keywords:** Machine learning, Predictive model, bacterial/fungal infection, Healthcare-associated, Nosocomial infection

## Abstract

**Background:**

COVID-19 and bacterial/fungal coinfections have posed significant challenges to human health. However, there is a lack of good tools for predicting coinfection risk to aid clinical work.

**Objective:**

We aimed to investigate the risk factors for bacterial/fungal coinfection among COVID-19 patients and to develop machine learning models to estimate the risk of coinfection.

**Methods:**

In this retrospective cohort study, we enrolled adult inpatients confirmed with COVID-19 in a tertiary hospital between January 1 and July 31, 2023, in China and collected baseline information at admission. All the data were randomly divided into a training set and a testing set at a ratio of 7:3. We developed the generalized linear and random forest models for coinfections in the training set and assessed the performance of the models in the testing set. Decision curve analysis was performed to evaluate the clinical applicability.

**Results:**

A total of 1244 patients were included in the training cohort with 62 healthcare-associated bacterial/fungal infections, while 534 were included in the testing cohort with 22 infections. We found that patients with comorbidities (diabetes, neurological disease) were at greater risk for coinfections than were those without comorbidities (OR = 2.78, 95%CI = 1.61–4.86; OR = 1.93, 95%CI = 1.11–3.35). An indwelling central venous catheter or urinary catheter was also associated with an increased risk (OR = 2.53, 95%CI = 1.39–4.64; OR = 2.28, 95%CI = 1.24–4.27) of coinfections. Patients with PCT > 0.5 ng/ml were 2.03 times (95%CI = 1.41–3.82) more likely to be infected. Interestingly, the risk of coinfection was also greater in patients with an IL-6 concentration < 10 pg/ml (OR = 1.69, 95%CI = 0.97–2.94). Patients with low baseline creatinine levels had a decreased risk of bacterial/fungal coinfections(OR = 0.40, 95%CI = 0.22–0.71). The generalized linear and random forest models demonstrated favorable receiver operating characteristic curves (ROC = 0.87, 95%CI = 0.80–0.94; ROC = 0.88, 95%CI = 0.82–0.93) with high accuracy, sensitivity and specificity of 0.86vs0.75, 0.82vs0.86, 0.87vs0.74, respectively. The corresponding calibration evaluation *P* statistics were 0.883 and 0.769.

**Conclusions:**

Our machine learning models achieved strong predictive ability and may be effective clinical decision-support tools for identifying COVID-19 patients at risk for bacterial/fungal coinfection and guiding antibiotic administration. The levels of cytokines, such as IL-6, may affect the status of bacterial/fungal coinfection.

## Background

Respiratory virus infections are associated with an increased risk of bacterial/fungal infections, especially in lower respiratory tract infections [1, 2]. Current studies have reported that the prevalence of healthcare-associated bacterial/fungal infections in patients with COVID-19 ranges from 3.6–32% [2–6]. Researchers [4, 6–8]have shown that COVID-19 and bacterial/fungal coinfections in those patients might contribute to worse outcomes, such as prolonged hospitalization and a higher mortality rate.

The long-term impacts of viral and bacterial/fungal coinfections on antimicrobial resistance are severe public problems [9]. It is difficult for clinicians to identify coinfections early because of similar symptoms and signs, thus leading to a high rate of inappropriate prescription [10–12]. Early empiric antibiotic use varied from 27 to 84% across different hospitals [10]. Two multicenter cohort studies [10, 11] showed that the proportions of bacterial coinfection were lower than 10%, while the proportions of early empirical antibiotics were as high as 60%. However, without bacterial coinfections, antibiotic overuse not only does not benefit patients but also accelerates the development of antimicrobial resistance.

Previous studies [5, 8, 12–14] have focused on the characteristics and risk factors for bacterial coinfection in patients with COVID-19. In the literature, several predictors, such as WBC count, PCT, CRP, steroid use, invasive ventilation, central venous catheter, urinary catheter, tocilizumab, length of stay, ICU admission, comorbidity, played significant roles in discriminating healthcare-associated bacterial coinfections [1, 2, 4, 5, 12, 14–16].

Recent studies [9, 11, 17] have used scientific statistical methods to estimate the risk of healthcare-associated bacterial coinfections in COVID-19 patients, instead of limiting the identification of risk factors. Estimating the probability of an individual developing healthcare-associated infections could aid in earlier intervention, such as prescribing antibiotics or providing appropriate patient care. Therefore, establishing accurate predictive models has practical significance for clinical work and is beneficial for identifying high-risk patients and preventing and controlling them precisely.

As machine learning (ML) is used for disease diagnosis or prognosis prediction, it is feasible to identify patients at high risk of bacterial coinfections [9, 11, 17]. Compared to traditional models, machine learning models have faster processors and smarter algorithms [18, 19]. Rapid progress in machine learning has provided opportunities for improved patient healthcare [20]. In this retrospective cohort study, we investigated the risk factors and established different ML models to predict the risk of healthcare-associated bacterial/fungal coinfections among inpatients with COVID-19.

## Method

### Inclusion and exclusion criteria

Inpatients who tested positive for COVID-19 according to nasopharyngeal swab PCR between January 1 and July 31, 2023 in a tertiary hospital in China were included. This hospital serves a population of more than nine million people and provides tertiary referral services to the surrounding regions. The exclusion criteria were as follows: [1] patients under 18 years of age [2], had a hospital stay less than three days, and [3] repeated patients.

### Definitions

According to the CDC/NHSN surveillance definition, healthcare-associated infections, also known as hospital-acquired infections, occur while receiving health care in the healthcare facility or hospital, are usually acquired ≥ 48 h after admission, and are not present or might be incubating on admission [21–26].

Healthcare-associated bacterial/fungal coinfections among COVID-19 inpatients: COVID-19 inpatients with signs of bacterial or fungal infection that develop 48 h after admission and have positive cultures are considered healthcare-associated bacterial/fungal coinfections. Our study excludes community-acquired infections [8].

Neurological diseases refer to disorders affecting the brain, spinal cord, and nerves throughout the body, including Parkinson’s disease, Alzheimer’s disease, multiple sclerosis, stroke, epilepsy, migraines, neuralgia, and various types of brain and spinal cord injuries.

### Study design and data collection

We have a real-time healthcare-associated infection surveillance system to monitor infections closely. Inpatients’ clinical information is recorded in the real-time surveillance system where clinicians and infection prevention and control professionals (IPCs) could receive early warnings about infections such as fever(> 38℃), elevated inflammatory markers(WBC or neutrophil count, PCT, IL-6, CRP), chest CT showing inflammation, antibiotic use or escalating antibiotic use, and positive cultures. Microbiological isolation is mandatory to confirm a bacterial/fungal infection. According to the symptoms and signs of the patient, clinicians will collect the specimens from suspected infection sites for etiological cultures, such as blood, urine, bronchoalveolar lavage(BAL), sputum, pleural fluid, ascites, and other specimens. Clinicians will diagnose and report healthcare-associated bacterial/fungal infections to the surveillance system. Meanwhile, IPCs will review medical record information to verify the occurrence or absence of infections. In summary, whether a healthcare-associated bacterial/fungal infection has occurred will be determined according to the symptoms and signs of patients and the culture-positive results of the suspected infection site. Based on the real-time surveillance system and microbiology culture, we can identify healthcare-associated bacterial/fungal infections as much as possible.

In this retrospective, single-center cohort study, data including demographic information, comorbidity information and laboratory results at admission were collected directly from the surveillance system. All predictive factors in our study preceded the outcome instead of a random point during the hospital stay. We also collected treatment information such as operation history, invasive ventilation, urinary catheter, meprednisone, dexamethasone, and tocilizumab before the infections occurred.

### Data processing and statistical analysis

All the data processing and analysis were conducted using R (version 4.3.0). Missing value were processed for weight (*n* = 477,26.83%), height (*n* = 387,27.77%), white blood cell count (*n* = 6,0.34%), lymphocyte count (*n* = 9,0.51%), PCT(*n* = 481,27.05%), CRP(*n* = 66,3.71%), IL-6(*n* = 561,31.5%), neutrophil (*n* = 6,0.34%), albumin(*n* = 40,2.25%), hemoglobin(*n* = 9,0.51%), creatinine(*n* = 82,4.61%), and glucose (*n* = 6,0.34%) according to multiple imputation method and were conducted for five imputations.

Continuous variables are reported as the medians and inter-quartile ranges (IQRs) and were compared using the Kruskal-Wallis test. Categorical variables are reported as counts and percentages and were compared using the Chi-sq or Fisher’s exact test. We conducted univariate and stepwise multivariate logistic regression analyses to investigate risk factors for healthcare-associated(HA) bacterial/fungal infection. Factors with a *P*-value less than 0.05 were independently associated with HA infections. Adjusted odds ratios (AORs) and 95% confidence intervals (95%CIs) were estimated.

### Model development and internal validation

We randomly divided all the samples into a training set and a testing set at a ratio of 7:3. The training set was used to screen variables and develop models, while the testing set was used for model evaluation. We selected variables for the model development which were statistically significant in our univariate analysis. The models included 14 candidate predictors, as follows: diabetes, kidney disease, neurological disease, ICU admission, PCT_level, albumin (ALB_level), creatinine (Cr_level), IL-6_level, CRP_level, neutrophil percent (Ne_level), central venous catheter (CVC), urinary catheter (UC), invasive ventilation (IV), and dexamethasone (DXM). The variance inflation factors (VIF) were calculated to assess the multicollinearity of the predictors. As all the predictors had a VIF less than 2, indicating no multicollinearity, all the predictors were included in the model development.

A random forest model was established (ntree = 500, mtry = 4) and the importance of the variables was determined. Our study compared the discrimination of models by the area under the receiver operating curve (AUCROC). The calibration slopes were calculated to check the risk of overfitting. Decision curve analyses were performed to evaluate whether the risk models improved clinical decision-making [27].

## Results

### Baseline characteristics

**Fig. 1:**
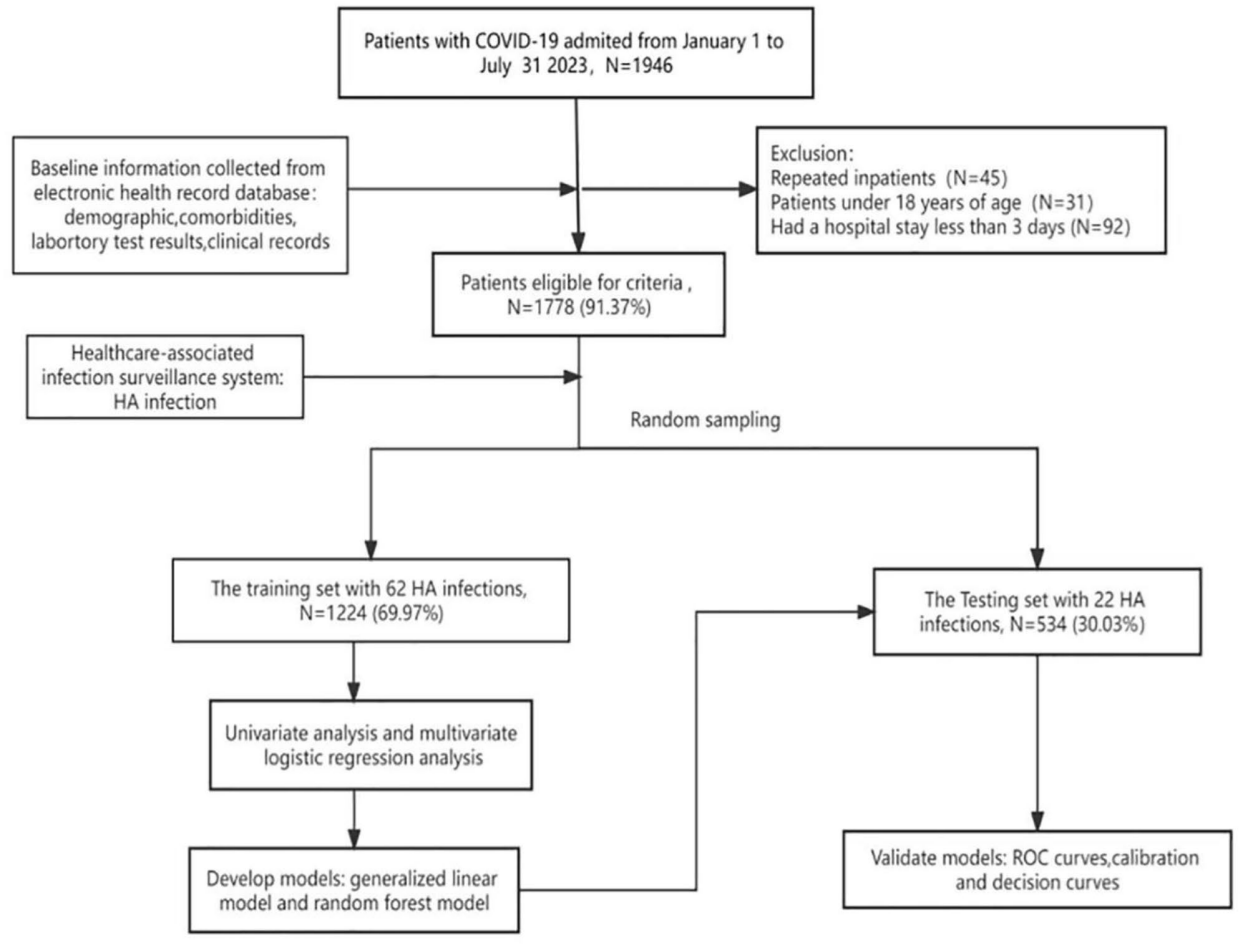
Flowchart of study participant selection and model development and validation

A total of 1946 inpatients were diagnosed with laboratory-confirmed with COVID-19 between January 1 and July 31, 2023. As shown in the Figs. 1 and 1778 eligible inpatients were enrolled in this study. The median age of the patients was 69 years (interquartile rage (IQR), 56–80 years), and 1043 were male (58.66%). The Table 1 shows the difference in baseline characteristics between the HA infection group and the Non-HA infection group. Eighty-four (4.72%) patients developed healthcare-associated bacterial/fungal infections, 75 of whom were bacterial infections and 9 of whom had fungal infections. The most common bacterial strain isolated was *klebsiella pneumoniae* which was found in 18 patients and the main infection site was the lower respiratory tract.

**Table 1:** Demographic characteristics, comorbidities, and laboratory test results for patients with HA bacterial/fungal infections and non-HA infections at baseline

Characteristics	Total (*N* = 1778)	HA infection (*N* = 84)	Non-HA infection (*N* = 1694)	c^2/W^	*P*
Gender [n,%]				0.08	0.78
male	1043(58.66)	51(60.71)	992(58.56)		
female	735(41.34)	33(39.29)	702(43.44)		
Age^*^, year [M, IQR]	69(56,80)	75(60,86.25)	68(56,79)	54,978	**< 0.001**
BMI^*^, kg/m^2^ [M, IQR]	23.39(21.20,25.95)	22.59(19.82,24.34)	23.43(21.25,26.03)	27,744	**0.04**
Hypertension [n,%]				1.34	0.25
Yes	896(50.39)	48(57.14)	848(50.06)		
No	882(46.61)	36(42.86)	846(49.94)		
Diabetes [n,%]				33.93	**< 0.001**
Yes	470(26.43)	47(55.95)	423(24.97)		
No	1308(73.56)	37(44.05)	1271(75.03)		
Tumor [n,%]				0.59	0.44
Yes	455(25.59)	25(29.76)	430(25.38)		
No	1323(74.41)	59(70.24)	1264(74.62)		
Kidney disease [n,%]				8.47	**0.004**
Yes	655(36.84)	44(52.38)	611(36.07)		
No	1123(63.16)	40(47.62)	1083(63.93)		
Neurological disease [n,%]				24.14	**< 0.001**
Yes	571(32.11)	48(57.14)	523(30.87)		
No	1207(67.89)	36(42.86)	1171(69.13)		
Operation[n,%]				0.09	0.76
Yes	351(19.74)	15(17.86)	336(19.83)		
No	1427(80.26)	69(82.14)	1358(80.17)		
ICU admission [n,%]:				11.24	**< 0.001**
Yes	111(6.24)	13(15.48)	98(5.79)		
No	1667(93.76)	71(84.52)	1596(94.21)		
Treatments before coinfections					
Invasive ventilation (IV)[n,%]				49.65	**< 0.001**
Yes	224(12.60)	32(38.10)	192(11.33)		
No	1554(87.40)	52(61.90)	1502(88.67)		
Urinary catheter (UC)[n,%]				58.08	**< 0.001**
Yes	566(31.83)	59(70.24)	507(29.93)		
No	1212(68.17)	25(29.76)	1187(70.07)		
Central venous catheter(CVC)[n,%]				53.34	**< 0.001**
Yes	433(24.35)	49(58.33)	384(22.67)		
No	1345(75.65)	35(41.67)	1310(77.33)		
Dexamethasone (DXM)[n,%]				21.69	**< 0.001**
Yes	537(30.20)	45(53.57)	492(29.04)		
No	1241(69.80)	39(46.43)	1202(70.96)		
Meprednisone(MEP) [n,%]				2.33	0.13
Yes	594(33.41)	35(41.67)	559(33.00)		
No	1184(66.59)	49(58.33)	1135(67.00)		
Tocilizumab^**^(TZ)[n,%]					1
Yes	21(1.18)	1(1.19)	20(1.18)		
No	1757(98.82)	83(98.81)	1674(98.81)		
Laboratory test results on admission					
White blood cell count(WBC)^*^, 10^9^/L[M, IQR]	6.20(4.70,8.50)	7.70(5.50,10.20)	6.10(4.70,8.40)	53,210	**< 0.001**
Neutrophil percent^*^,% [M, IQR]	70.21(59.80,82.20)	79.7(67.60,90.50)	70.6(59.50,81.60)	47,654	**3.818e-07**
Lymphocyte count(Lym)^*^, [M, IQR]	1.10(0.70,1.60)	0.90(0.58,1.33)	1.1(0.70,1.60)	83,667	**0.005**
PCT^*^, ng/ml[M, IQR]	0.07(0.04,0.25)	0.14(0.07,1.50)	0.071(0.04,0.20)	34,758	**4.473e-06**
IL-6^*^,pg/ml[M, IQR]	22.52(7.49,54.31)	18.02(5.54,42.82)	22.67(7.69,56.10)	33,935	0.14
CRP^*^, mg/L[M, IQR]	12.10(4.10,47.88)	50.30(10.50,114.15)	10.90(3.90,45.30)	44,401	**1.936e-07**
Albumin^*^(ALB), g/L [M, IQR]	36.20(52.00,39.20)	33.00(29.10,35.88)	36.3(32.60,39.30)	94,897	**1.452e-08**
Creatinine(Cr)^*^, umol/L[M, IQR]	65.00(52.00,86.90)	75.00(54.75,110.00)	65.00(52.00, 85.18)	61,231	**0.07146**
Hemoglobin(Hb)^*^, g/L [M, IQR]	120(104,133)	112.5(95.75,126.50)	120(104,133)	80,214	**0.04**
Glucose(Glu)^*^, mmol/L [M, IQR]	6.584(4.52,7.29)	6.705(5.01,10.69)	5.26(4.50,7.14)	48,508	**5.007e-06**
Length of hospital stay^*^, day [M, IQR]	13.12(7.00,16.00)	13.00(6.00,19.00)	11.00(7.00,16.00)	68,409	0.55
Neutrophil and lymphocyte ratio(NLR)^*^,[M, IQR]	3.73(2.08,7.61)	6.25(2.83,17.45)	3.69(2.06,7.43)	51,301	**2.036e-05**
Ferritin^*^(Fe), ng/ml[M, IQR]	366.4(185.5,710.7)	491.80(343.25,1036.7)	355.7(173.15,690.95)	11,922	**< 0.001**

The bold values indicate that these factors were statistically significant

M: median, IQR: interquartile range

* The statistical analysis were performed with the Kruskal-Wallis test

** *P*-value calculated by Fisher’s exact probability method

According to random sampling results, a total of 1244 patients in the training set had 62 HA infections, while 534 patients in the testing set had 22 HA infections. There was no significant difference in the HA infection rate between the two groups (*P* = 0.51).

**Table 2:** Sampling results for the training set and testing set

Datasets	HA infection	Non-HA infection	Total	c^2^	*P*
The training set	62(4.98%)	1182(95.02%)	1244(69.97%)	0.44	0.51
The testing set	22(4.12%)	512(95.88%)	534(30.03%)		
Total	84(4.72%)	1694(95.28%)	1778(100.00%)		

## Model development

### General linear model

The result of the ANOVA test (*P* = 0.66) indicated no significant difference between the full and stepwise models, and the AIC of the stepwise model was lower (417.22) than that of the full model (426.17). Thus, the stepwise logistic regression model was chosen as the final general linear model and included 7 predictors, as shown in Table 3.

### Independent risk factors

According to the univariate analysis, 14 variables were associated with healthcare-associated bacterial/fungal infection, including diabetes, kidney disease (SZB), neurological disease (SJB), invasive ventilation (IV), urinary catheter (UC), central venous catheter (CVC), ICU admission, IL-6_level < 10pg/ml, CRP_level < 10 ng/ml, PCT_level > 0.5 ng/ml, Cr_level < 44 umol/L, Ne_level < 80%, Lym_level < 0.2 × 10^9^/L, and dexamethasone (DXM) (*P* < 0.05). These factors were subsequently inputted during the model development.

As shown in Table 3, compared with patients without diabetes, patients with diabetes had a 2.78-fold increase (95%CI = 1.61–4.86) in the risk of being infected. Patients with neurological disease (AOR = 1.93, 95%CI = 1.11–3.35), CVC (AOR = 2.53, 95%CI = 1.39–4.64) or UC (AOR = 2.28, 95%CI = 1.24–4.27) were more likely to be infected. A PCT concentration>0.5 ng/ml(AOR = 2.03, 95%CI = 1.41–3.82) was associated with increased risk. Cr<44 umol/L (AOR = 0.40, 95%CI = 0.22–0.71) was a protective factor. An IL-6 concentration < 10 pg/ml might be associated with increased infection risk (AOR = 1.69, 95%CI = 0.97–2.94).

**Table 3:** Univariate and multivariate logistic regression analyses with the stepwise method in the training set (*n* = 1244)

Characteristics	Total(%)	Univariate analysis			Multivariate regression	
		COR (95%CI)	*P*		AOR (95%CI)	*P*
Gender, male	510(40.99)	1.19(0.68–2.14)	0.54			
Age group,<65year	509(40.92)	0.73(0.40–1.30)	0.30			
BMI_level<30	751(60.37)	0.88(0.35–2.97)	0.81			
Hypertension	611(49.12)	0.92(0.53–1.60)	0.76			
Diabetes	344(27.65)	4.11(2.36–7.29)	**8.09e-07**		2.78(1.61–4.86)	0.0002
Tumor	307(24.68)	1.35(0.73–2.40)	0.32			
Kidney disease	443(35.61)	1.93(1.11–3.38)	**0.02**			
Neurological disease	389(31.27)	2.07(1.19–3.61)	**0.01**		1.93(1.11–3.35)	0.02
Invasive ventilation	153(12.29)	3.57(1.91–6.44)	**< 0.001**			
Urinary catheter	393(31.59)	4.64(2.62–8.47)	**< 0.001**		2.28(1.24–4.27)	0.01
Central venous catheter	290(23.31)	5.10 (2.91–9.11)	**1.76e-08**		2.53(1.39–4.64)	0.002
Operation	243(19.53)	0.92(0.43–1.78)	0.81			
ICU admission	77(6.19)	2.44(0.98–5.27)	**0.04**			
IL-6_level<10 pg/ml	388(31.19)	1.75(1.04–2.94)	**0.03**		1.69(0.97–2.94)	0.06
CRP_level<10 mg/L	589(47.35)	0.35(0.18–0.63)	**< 0.001**			
PCT_level > 0.5 ng/ml	163(13.10)	4.00(2.33–7.14)	**< 0.001**		2.03(1.41–3.82)	0.03
Cr_level<44 umol/L	907(72.91)	0.29(0.14–0.43)	**< 0.001**		0.40(0.22–0.71)	0.002
WBC_level<9.5 × 10^9^/L	988(79.42)	0.63 (0.34–1.22)	0.15			
Ne_level < 80%	859(69.05)	0.40(0.24–0.67)	**0.0005**			
Lym_level<0.2 × 10^9^/L	6(0.48)	5.71(0.29–39.42)	0.12			
ALB_level<35 g/L	513(41.24)	2.85(1.62–5.20)	**0.0004**			
Hb_level<120 g/L	601(48.31)	1.15(0.69–1.92)	0.59			
Dexamethasone(DXM)	368(29.58)	2.59(1.49–4.52)	**0.001**			
Meprednisone (MEP)	430(34.57)	1.46(0.83–2.55)	0.18			
Tocilizumab(TZ)	14(1.12)	1.74(1.00–9.00)	0.60			
Length of hospital stay<7days	263(21.14)	1.09(0.57–1.96)	0.78			

Ref: reference

### Random forest model

**Fig. 2:**
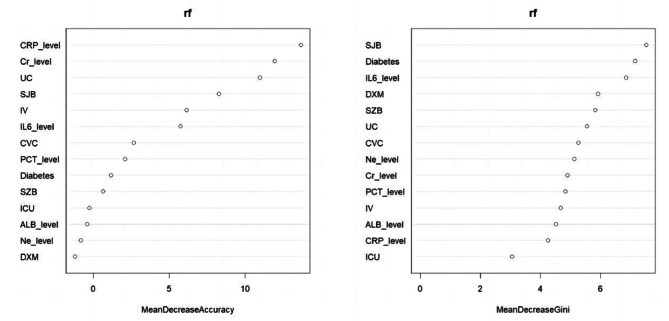
Variable importance for the random forest model (RFM). SZB, kidney disease; SJB, neurological disease; ICU, ICU admission; ALB_level, albumin level; Cr_level, creatinine level; Ne_level, neutrophil level; CVC, central venous catheter; UC, urinary catheter; IV, invasive ventilation; DXM, dexamethasone

The RF model was trained using 1244 inpatients and 14 variables. The random forest model yielded an out-of-bag error of 4.98%. As shown in Fig. 2, the importance of the variables was obtained as follows: using the mean decrease in Gini as a criterion, neurological disease, diabetes, IL-6 levels and dexamethasone made the greatest contributions.

## Model performance and comparison

### Discrimination

**Fig. 3:**
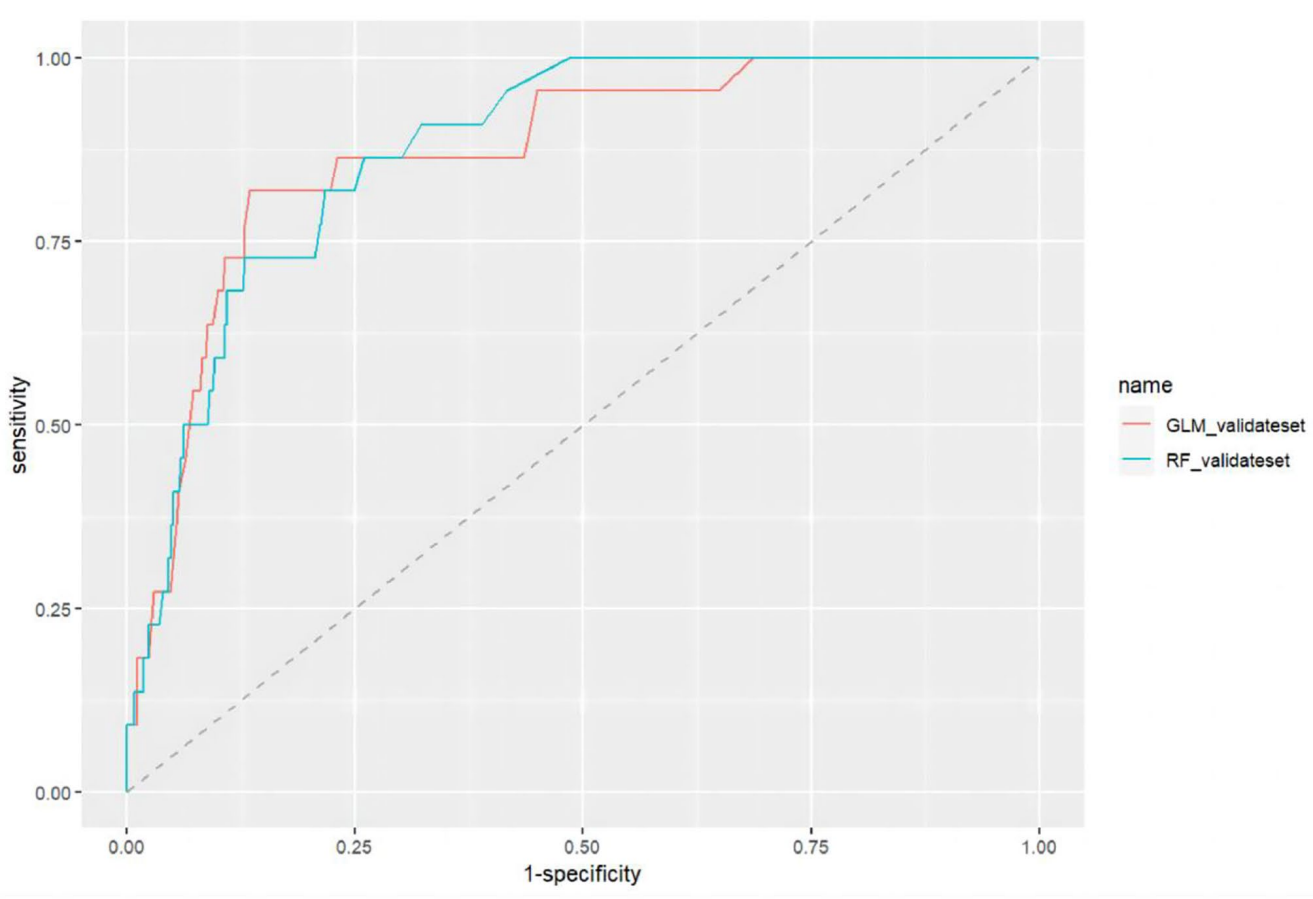
Performance of different machine learning models(the testing set, *n* = 534)

The two different models achieved comparable performance levels, as shown in Fig. 3. The AUCROCs for the GLM and RFM were 0.87(95%CI = 0.80–0.94) and 0.88(95%CI = 0.82–0.93), respectively. The RFM slightly outperformed than the GLM. The sensitivities of both models were greater than 80%.

**Table 4:** Statistics and classification matrix of the testing set

Models	ROC(95%CI)	Cutoff	TP	TN	FP	FN	Sen	Spec	Acc	PPV	NPV	F1-score
GLM	0.87(0.80–0.94)	0.069	18	443	69	4	0.82	0.87	0.86	0.21	0.99	0.33
RFM	0.88(0.82–0.93)	0.023	19	379	133	3	0.86	0.74	0.75	0.13	0.99	0.22

Sen sensitivity, Spec specificity, TN true negative, FN false negative, TP true positive, FP false positive, PPV positive predict value, NPV negative predict value

Sens(Recall) = TP/(TP + FN)

Spec = TN/(TN + FP)

PPV (Precision) = TP/(TP + FP)

Acc=(TP + TN)/(TP + FP + TN + FN)

F1-score = 2*(Precision*Recall)/(Precision + Recall)

### Calibration

**Fig. 4:**
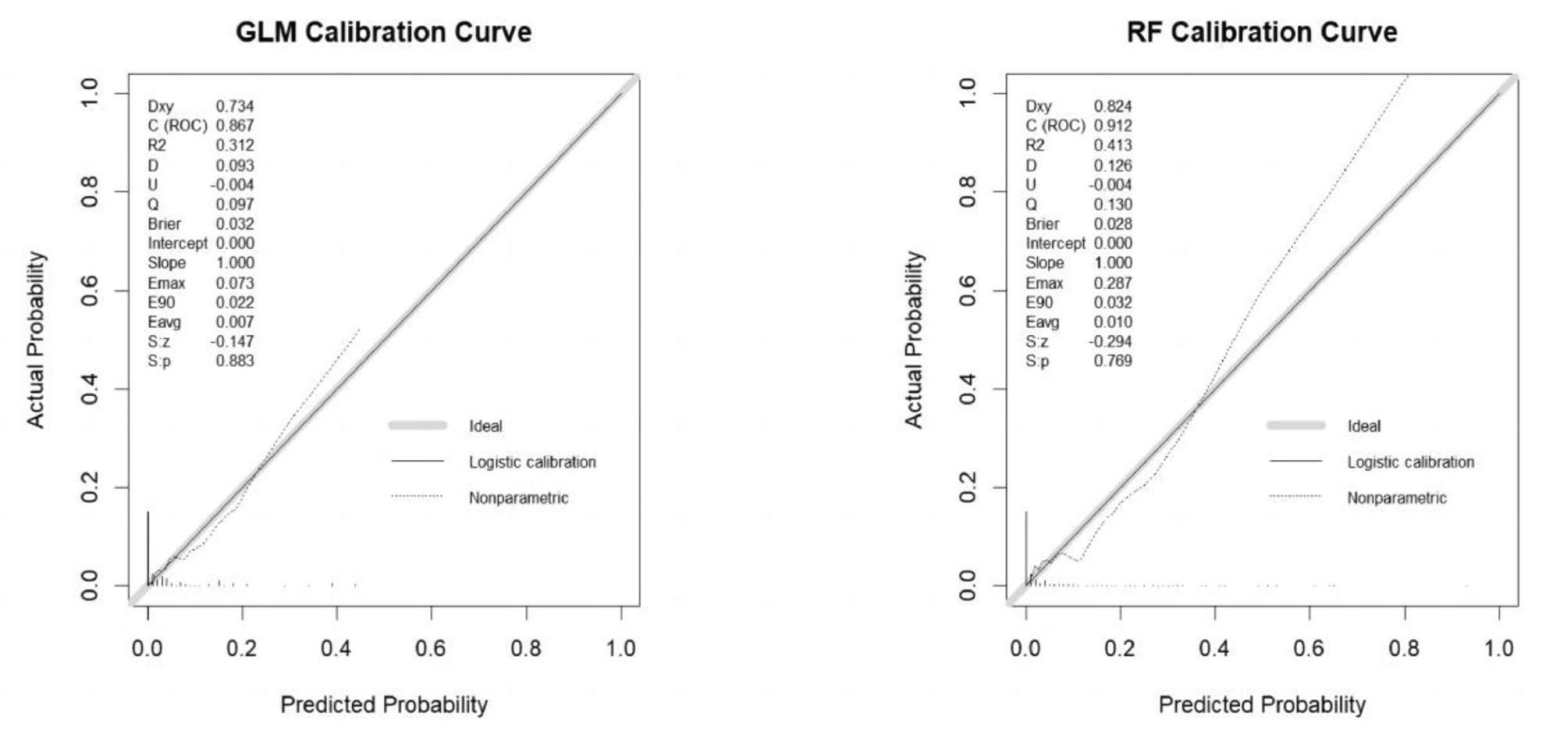
Calibration curves of different machine learning models (the testing set, *n* = 534)

As shown in Fig. 4, the calibration lines were close to the ideal lines, and a slope of 1 indicated no overfitting. The Dxy over 0.7 indicated good correlations between the predictive and actual values, which showed that RFM was better than GLM(0.824vs0.734). The mean square error(Brier) of GLM and RFM were 0.032 and 0.028, respectively, and the smaller the better. The S: p was the *P* value(> 0.05) of the Z test, which indicated the fitness effects were relatively excellent. Those indicators in the two models were closed, but the calibration of RFM outperformed slightly than that of GLM.

### Decision curve

**Fig. 5:**
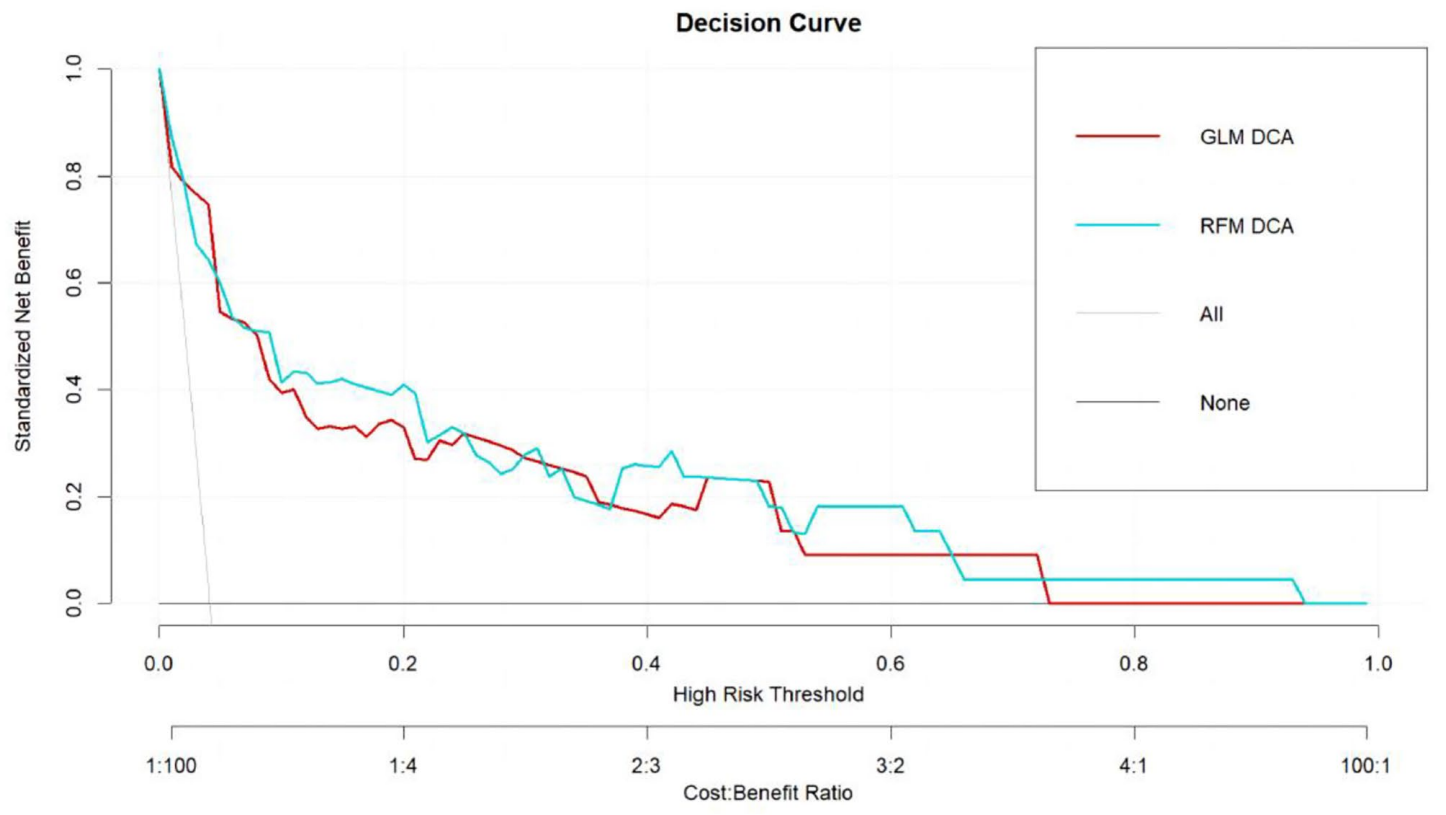
Decision curves for the default strategies and for GLM and RFM(the testing set, *n* = 534)

As shown in Fig. 5, both models had greater standard net benefits than default strategies across the threshold range. Thus, both models had better utility in supporting clinical decisions and led to the best decisions.

## Discussion

Bacterial/fungal coinfection is a serious complication of COVID-19, especially in the presence of comorbidities, and can lead to a worse prognosis and antibiotic overuse [28]. In the present study, of a total of 1778 patients hospitalized with COVID-19, approximately 5% presented with bacterial/fungal coinfections. We has investigated the risk factors associated with bacterial/fungal infections and developed machine learning-based models with robust predictive performance. The algorithm showed that comorbidities (diabetes, neurological diseases), invasive procedures (central venous catheter, urinary catheter), baseline inflammatory markers levels (IL-6, PCT), and creatinine were associated with an increased risk of bacterial/fungal infection. Those predictors are less expensive, faster, and easier to obtain from electronic medical records. The machine learning-based models are preferred methods for infection surveillance and disease prognosis, which makes it easier to identify high-risk inpatients. When the estimated coinfection risk is low, it is recommended to limit or use antibiotics cautiously, whereas high-risk estimates suggest enhancing surveillance or resource reallocation through additional patient care or enhanced disinfection, which could improve the efficiency of hospital infection surveillance [29]. Early detection of high-risk patients is beneficial for preventing hospital infection outbreaks, antibiotic overuse, and microbial resistance.

Diabetes is related to various infections, especially skin, lower respiratory tract, and urinary tract infections [30]. A review suggested that diabetes and its comorbidity may lead to some infectious diseases due to metabolic disturbances [30]. Similarly, Suheda Erener [31] summarized the clinical data showing that diabetes and neurological disease may render patients more vulnerable to infectious diseases. In line with the findings of previous studies [2, 12], multivariate logistic analysis indicated that central venous and urinary catheters are associated with increased infection risk. The most common infection source of catheters is intradermal and catheter interface contamination by organisms, which may come from the patient’s skin or from healthcare workers’ hands. Patients with catheters have severe disease and lower immunity, so it is harder to defend against bacterial invasion. In our study, these factors were inputted as strong predictors for model development which gained promising results for risk estimates.

PCT is a well-known biomarker of bacterial infection and is involved in the early recognition of bacterial coinfection in patients with influenza pneumonia. Several studies have noted that high PCT levels on admission are associated with severe outcomes in critically ill patients [28, 32]. We found that PCT > 0.5 ng/ml was associated with an increased coinfection risk, which had a significant predictive value for bacterial/fungal coinfection among COVID-19 patients. Similarly, a study reported that a PCT cut-off value at 0.55ng/mL on admission may help identify bacterial coinfections [33]. However, a meta-analysis concluded that PCT has limited predictive value for bacterial coinfections, but lower PCT levels might indicate a decreased risk [34]. Although the value of PCT in predicting bacterial coinfection in patients has remained controversial, a continuous increase in PCT levels may indicate bacterial coinfections and progression toward more severe complications [35–37]. Nonetheless, clinicians could consider not administrating antibiotics in patients with a PCT level lower than 0.5 ng/ml, which could be a helpful decision-support tool to guide antibiotic therapies for COVID-19 [33, 38, 39].

IL-6 is a prototypical cytokine with pleiotropic activity that contributes to maintaining homeostasis [40]. Previous reports have investigated that an acute infection response induces rapid production of IL-6, which activates the host defense mechanism against infection through elevated acute-phase proteins and the immune response [40, 41]. In our study, a level of IL-6 lower than 10 pg/mL may indicate bacterial/fungal coinfections, likely due to immunosuppression or corticosteroid therapy in the hospital. If the produced IL-6 level is deficient at the acute infection response phase, the host might not defend against secondary infections. However, excessive IL-6 levels and uncontrolled IL-6 receptor signaling are common in critically ill patients [42]. By being vigilant and monitoring IL-6 levels, healthcare professionals can identify potential coinfections and provide appropriate treatment, ultimately improving patient outcomes. Cytokine storm, exacerbation synthesis of cytokines, can deteriorate the patient’s clinical conditions [43]. Future studies could explore cytokine levels and changes at different phases in bacterial/fungal coinfection and their impact on prognosis among COVID-19 patients.

Creatinine is a biomarker of kidney function. Several studies evaluated the association between biomarkers of abnormal kidney disease and death in COVID-19 patients, which found that patients with increased creatinine or low glomerular filtration rate at baseline had a poor prognosis [44, 45]. Our study pointed out that patients with low creatinine levels at baseline had a decreased risk of bacterial/fungal coinfections, which possibly because acute kidney function injury has not yet occurred. However, the relationship between kidney disease and post-acute COVID-19 syndrome is not yet determined, and prospective studies need to measure more laboratory biomarkers, such as glomerular filtration rate and urinary β^2^-microglobulin, to assess kidney function [46].

In summary, these factors are invaluable in accurately predicting and assessing the risk of bacterial/fungal coinfections. Incorporating them into our models not only enables us to make informed decisions but also helps us take proactive measures to prevent such infections.

Recent studies have initiated the prediction models to identify bacterial coinfections among CPVID-19 patients. A study [11] in Italy calculated a predictive risk score by assigning a point value according to the β coefficient to classify patients at risk of bacterial coinfection. This intuitive approach may be useful in diagnostic testing and antibiotic use. Machine-learning(ML) algorithms are novel and rapidly evolving technologies providing opportunities for clinical decision support in healthcare [11]. RAWSON T M et al. [9] have demonstrated that a support vector machine (SVM) with 21 blood test variables can accurately predict positive microbiological samples. However, it’s important to note that the study only focused on comparing algorithm performance and piloting the algorithm on a small group of patients who were admitted to the hospital. Ferentzakis et al. [47]have conducted five ML techniques to explore the association rules in antimicrobial resistance profiles in the ICU. They have forecast antimicrobial resistance of Acinetobacter baumannii, Klebsiella pneumoniae, and Pseudomonas aeruginosa, which could be a low-cost decision-support tool in selecting the appropriate empirical antibiotic treatment [48]. Another study [29] has developed ML models for the surveillance of surgical site infections(SSI), which demonstrated that ML could improve the efficiency of SSI surveillance by decreasing the burden of chart review with high sensitivity.

Discrimination is a traditional performance metric in model evaluation that uses the AUCROC or C statistic to compare models. In our study, the AUCROCs of the two models exceed 0.85 with excellent discrimination, which indicated those models well differentiated high-risk groups from those at lower risk. However, discrimination alone is insufficient to assess the performance of predictive models, and calibration or goodness of fit is often regarded as most reliable property of a model [49]. Few studies have drawn calibration curves to evaluate the matching degree between predicted and actual probabilities [20]. Our calibration lines were close to the ideal calibration line. Both slopes were approximately equal to 1, and the intercepts were equal to 0, indicating no overfitting, overestimates, or underestimates of our models. The Dxy indicated the correlations between the predictive and actual values, which showed that RFM was better than GLM(0.824vs0.734). The mean square error(Brier) of GLM and RFM were 0.032 and 0.028, respectively, the smaller the better. So, the calibration of RFM outperformed slightly than that of GLM. The decision curves showed that these models had greater standard net benefits across all risk thresholds, which indicated that early management of high-risk patients could be beneficial according to our models [20]. In summary, we should combine multiple measures to evaluate the pros and cons of models.

Our study has several limitations. First, we may underestimate the prevalence of bacterial/fungal infections. Generally, clinicians and IPCs diagnose and report healthcare-associated infection cases, and the number of cases detected partly relies on the extent of their efforts and the sensitivities of surveillance. Some infections might not be included due to the low culture-positive rate such as blood and cerebrospinal fluid samples. Second, some indicators, heart failure, cirrhosis, chronic kidney disease(CKD), glomerular filtration rate(GFR), ferritin, and suPAR levels, which may be associated with the prognosis of COVID-19 infection, have not been selected as the candidate predictors due to the retrospective study design. In the future, prospective and multi-center studies can directly measure more parameters to improve and externally validate the predicting models. Third, we did not test other viral infections, but viral coinfections are also significant to the prognosis of COVID-19 patients. However, identifying the risk factors of bacterial/fungal coinfections and estimating the probability of coinfections could guide the rational use of antibiotics.

## Conclusions

Our results indicate that the machine learning models achieved strong predictive ability and may be effective clinical decision-support tools for bacterial/fungal infection surveillance and for guiding antibiotic administration. The GLM suggested that patients with an IL-6 concentration < 10pg/ml are more vulnerable to developing a bacterial/fungal infection.

## Data Availability

The datasets used and/or analyzed during the current study are available from the corresponding author upon reasonable request.
